# Cyclosporine a in the treatment of dry eye disease: a narrative review

**DOI:** 10.3389/fopht.2025.1700163

**Published:** 2025-11-19

**Authors:** Xiaoyan Bian, Jun Ma, Yunxia Liu, Yuelan Feng, Zhiqiang Liu, Bozhou Zhang, Baoyu Huang

**Affiliations:** 1 Department of Corneal Diseases, Baotou Chaoju Eye Hospital, Baotou, China; 2 Department of Ophthalmology, Benxi Central Hospital, Benxi, Liaoning, China; 3 Department of Ophthalmology, The First Affiliated Hospital of Baotou Medical College of Inner Mongolia University of Science and Technology, Baotou, China; 4 Department of Ophthalmology, Yantai Huaxia Kang’ai Ophthalmic Hospital, Yantai, China; 5 Department of Ophthalmology, The First Affiliated Hospital of Guangxi Medical University, Nanning, China

**Keywords:** dry eye disease, cyclosporine A, inflammation, immune modulation, review

## Abstract

Dry eye disease (DED) is a common chronic ocular surface disorder that significantly impacts quality of life. Its pathogenesis involves disruption of immune regulatory mechanisms and ocular surface inflammation, which mutually reinforce each other in a vicious cycle. Conventional treatments, such as artificial tears and meibomian gland care, alleviate symptoms but often fail to control underlying inflammation. Anti-inflammatory therapy is therefore crucial. Traditional agents like corticosteroids provide rapid relief but carry risks with long-term use. Cyclosporine A, an immunosuppressant, offers unique advantages by inhibiting T-cell activation, reducing pro-inflammatory cytokines, enhancing tear secretion, and restoring the ocular surface. Clinical and experimental studies have consistently demonstrated its efficacy and safety in improving tear production, relieving symptoms, repairing ocular surface structures, and slowing disease progression. This review summarizes the mechanisms, recent clinical evidence, and future perspectives of topical cyclosporine A in DED treatment, providing a reference for rational clinical use and novel therapeutic development.

## Introduction

Dry eye disease (DED) is a common multifactorial disorder of the ocular surface. The pathophysiological mechanisms of DED include tear film instability and shortened tear break-up time (TBUT), tear hyperosmolarity, ocular surface inflammation and damage, and neurosensory abnormalities (1). In 2007, the International Dry Eye Workshop (DEWS I) first defined DED and identified inflammation as one of its major pathogenic mechanisms (2, 3).

Any insult to ocular surface function, such as reduced tear secretion, increased tear evaporation, or environmental irritation, can lead to tear film instability and elevated tear osmolarity, which in turn activate inflammatory cells on the ocular surface and trigger inflammatory cascades (4, 5). Consequently, controlling ocular surface inflammation and restoring microenvironmental stability are considered key strategies for DED management.

Current first-line treatments, including artificial tear supplementation, meibomian gland massage, warm compresses, and lifestyle modifications, can relieve symptoms to some extent but fail to fundamentally suppress the inflammatory process. Traditional anti-inflammatory agents, such as corticosteroids and nonsteroidal anti-inflammatory drugs, can rapidly improve symptoms but are unsuitable for long-term use due to adverse effects including intraocular pressure elevation, cataract formation, and corneal epithelial toxicity (6).

In recent years, emerging anti-inflammatory therapies such as lifitegrast, tacrolimus, and biologics targeting interleukin pathways have broadened the therapeutic landscape. Placing Cyclosporine A (CsA) within this context allows a clearer understanding of its advantages, such as long-term tolerability and immune-modulating specificity, as well as its limitations in onset speed and patient adherence (7). The latest TFOS DEWS II report further emphasized the role of inflammation in DED pathogenesis and recommended topical CsA as a long-term therapeutic option (6). Therefore, a comprehensive review of the mechanisms of action and clinical evidence of CsA in DED may provide valuable guidance for optimizing treatment strategies and improving patient outcomes.

This review aimed to provide an updated and integrative overview of the pharmacological mechanisms, formulation development, and clinical efficacy of topical CsA in the treatment of DED. Unlike earlier reviews that mainly summarized early clinical experiences, this work emphasizes recent evidence, comparative perspectives with other anti-inflammatory agents, and future research directions.

## Literature search strategy

A comprehensive literature search was conducted in PubMed, Embase, and Web of Science from January 2000 to August 2025 using the keywords “dry eye disease,” “cyclosporine A,” “ocular surface,” “formulation,” and “clinical trial.” Randomized controlled trials, cohort studies, and relevant experimental investigations were included. Reviews, case reports, and conference abstracts were excluded. Reference lists of retrieved articles were manually screened to identify additional studies.

### Mechanisms of CsA in the treatment of DED

**Figure 1:**
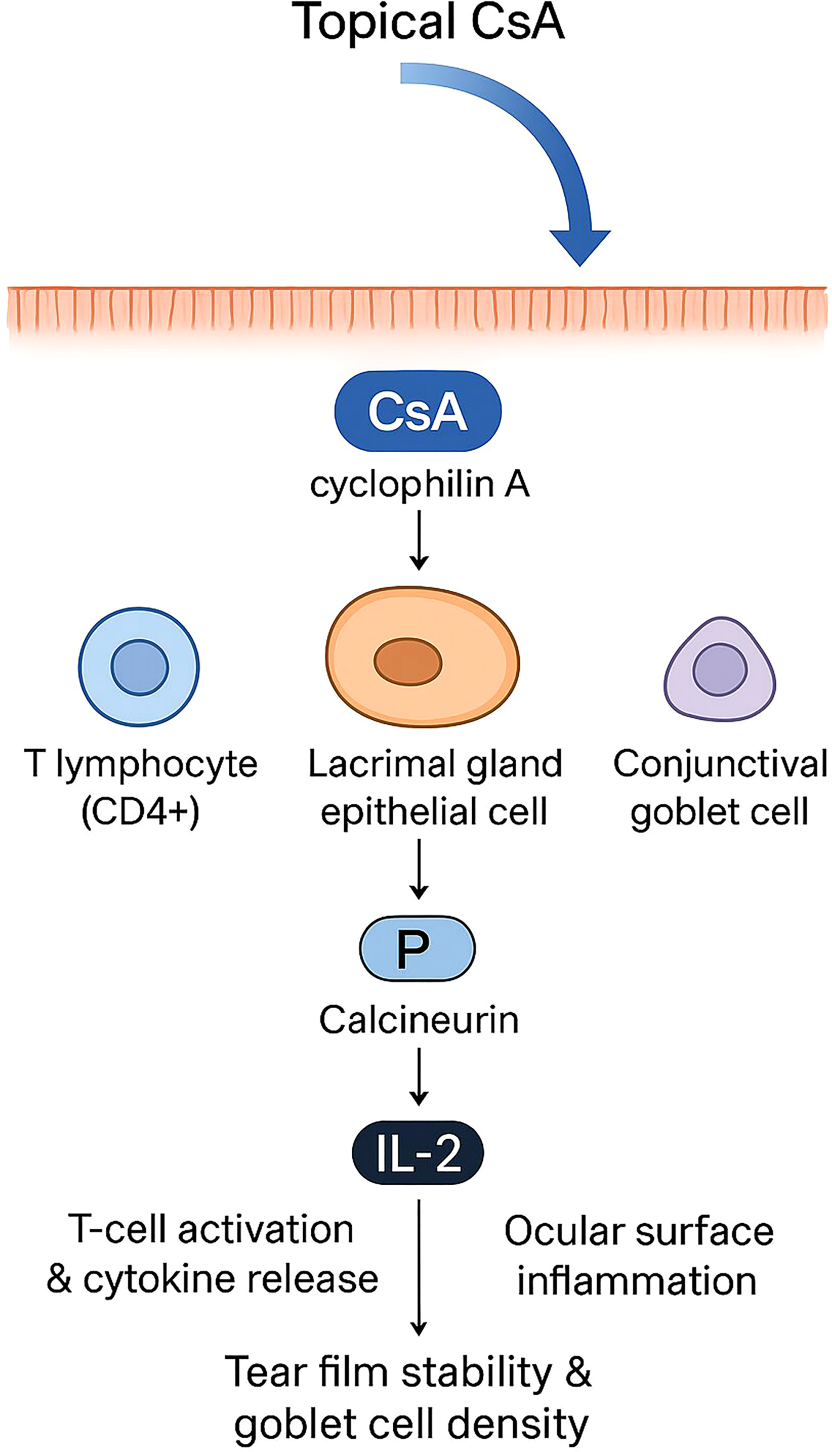
Mechanism of action of topical cyclosporine A (CsA) in dry eye disease. Topically applied CsA penetrates the ocular surface and binds to cyclophilin A within lacrimal gland epithelial cells and T lymphocytes. The CsA–cyclophilin A complex inhibits calcineurin activity, leading to decreased interleukin-2 (IL-2) transcription and suppression of T-cell activation and cytokine release. This cascade reduces ocular surface inflammation, stabilizes the tear film, and increases conjunctival goblet cell density, thereby improving the ocular surface microenvironment in dry eye disease.

CsA, a well-characterized immunosuppressant, inhibits T-cell activation, reduces inflammatory mediator release, and improves lacrimal gland function, thereby effectively alleviating DED signs and symptoms with favorable long-term safety. In addition, CsA is a cyclic, non-ribosomal neutral peptide with a molecular weight of 1,202 g/mol, marked hydrophobicity (LogP = 1.4–3.0), and extremely low aqueous solubility (6.6×10^6^ μg/ml, temperature-dependent), with only 0.008 mg/ml solubility in water at room temperature. These physicochemical properties limit its ocular delivery and bioavailability in topical formulations (**Figure 1**) (8).

At the cellular level, CsA penetrates the cytoplasm of T cells and binds to cyclophilin A, thereby inhibiting calcineurin phosphatase activity and blocking the transcription of IL-2 and other T cell activation-related genes (8). Additionally, CsA can bind to cyclophilin D to inhibit apoptosis or programmed cell death (8, 9), and suppress T cell–mediated inflammatory responses by blocking p38 and JNK signaling pathways. In the inflammatory milieu of DED, levels of inflammatory mediators such as IL-1, IL-2, IL-6, tumor necrosis factor-α (TNF-α), and matrix metalloproteinases (MMPs) are markedly elevated in tears and conjunctival epithelial cells. These mediators activate dendritic cells and recruit T lymphocytes, which further release cytokines, impair lacrimal functional unit function, damage corneal and conjunctival goblet cells, reduce mucin secretion, and amplify apoptosis and inflammation, thus perpetuating the vicious inflammatory cycle. CsA can effectively disrupt this cycle, reduce ocular surface T lymphocyte infiltration and inflammatory cytokine production, improve corneal and conjunctival epithelial apoptosis (10, 11), and significantly increase goblet cell density and function (12).

CsA also restores tear secretion suppressed by inflammation, a process that may not only depend on inflammation resolution but also involve stimulation of muscarinic and sensory nerves to release neurotransmitters (13, 14). In DED, MMP concentrations and activity in tears are elevated (15), leading to degradation of basement membrane components and tight junction proteins such as ZO-1 and occluding (16, 17), thereby weakening the corneal barrier. CsA treatment reduces MMP levels, helping repair and maintain ocular surface barrier integrity. In terms of mucin metabolism, CsA increases the proportion of secretory goblet cells and enhances intracellular mucin content, enabling faster mucin synthesis and accumulation compared to untreated conditions (18).

Furthermore, *in vivo* confocal microscopy studies have shown that CsA can reverse DED-induced activation of corneal Langerhans cells, loss of subbasal corneal nerve fiber density, and increased nerve tortuosity, while restoring corneal sensitivity (19, 20). Recovery of corneal nerve function can reduce the release of neuropeptides associated with neurogenic inflammation and even strengthen reflex arcs to stimulate blinking and tear secretion, thereby lowering tear osmolarity and creating favorable conditions for corneal nerve regeneration (20). Therefore, even patients with mild ocular surface inflammation may benefit from the multifaceted mechanisms of CsA.

### Clinical formulations of CsA

CsA has demonstrated definite efficacy and low toxicity in the treatment of DED. However, its large molecular weight, hydrophobicity, and low permeability limit the optimization of drug concentration, stability, and administration routes. To improve water solubility and ocular permeability, the concept of “nanoemulsion” was proposed, which consists of oil, surfactant, and co-surfactant, and is more easily absorbed than traditional emulsions (**Table 1**).

**Table 1:** Comparison of commercially available topical cyclosporine A formulations for dry eye disease.

Name	Location	CsA concentration	Vehicle/delivery system	Key features	Dosing frequency	Advantages	Limitations
Restasis^®^	Allergan, Irvine, CA, USA	0.05%	Anionic castor oil-in-water emulsion	First approved CsA ophthalmic emulsion (2002, FDA)	BID	Proven long-term efficacy	Delayed onset; stinging/burning
Ikervis^®^	Santen Pharmaceutical Co., Ltd., Osaka, Japan	0.10%	Cationic emulsion (Novasorb^®^ technology)	Enhanced corneal penetration	QD	Better tolerability, higher CsA concentration	May cause transient irritation
Cequa™	Sun Pharmaceutical Industries Ltd., Mumbai, India	0.09%	Nanomicellar formulation	Improved bioavailability & comfort	BID	Faster onset, improved stability	Cost; limited long-term data
CyclASol^®^	Novaliq GmbH, Heidelberg, Germany	0.10%	Water-free semifluorinated alkane (SFA) solution	Novel non-aqueous delivery; preservative-free	BID	Rapid symptom relief; non-blurring	Still under long-term evaluation
Cycloome^®^	Xingqi Pharmaceutical Co., Ltd. Shenyang, China	0.05%	Polyvinylpyrrolidone (PVP) aqueous solution	Immunomodulatory effect and long-term safety profile	BID	Improves symptoms of dry eye and lower risk of systemic side effects	Possible local ocular irritation

In 2002, the U.S. FDA approved Restasis^®^ (Allergan, Irvine, CA, USA), a 0.05% CsA anionic oil-in-water nanoemulsion for the treatment of inflammation-related tear secretion reduction, with a particle size of approximately 159.3 nm. Moreover, its oil-based high viscosity and poor stability often cause blurred vision, burning sensation, and conjunctival hyperemia, leading to poor tolerability and low bioavailability (21, 22). In China, Zirun^®^ (Xingqi Pharmaceutical Co., Ltd., Shenyang, China) (0.05% CsA clear nano-microemulsion, particle size about 24.2 nm) can show efficacy within 3 months, with blurred vision being rarely reported as an adverse event (23).

Ikervis^®^ (Santen Pharmaceutical Co., Ltd., Osaka, Japan), a 0.1% CsA cationic oil-in-water nanoemulsion, was approved by the European Union in 2015. By electrostatically interacting with the mucin layer, it prolongs retention time on the ocular surface, thereby improving bioavailability. Additionally, cationic emulsions themselves may promote tear secretion and modulate inflammatory cytokine expression (24, 25). In 2018, the FDA approved OTX-101 (Cequa™; Sun Pharmaceutical Industries Ltd., Mumbai, India), a 0.09% CsA nanomicellar aqueous solution, with a particle size of 10–80 nm. It can easily diffuse through scleral pores, with CsA concentrations in the conjunctiva and sclera being 3.60-fold and 3.44-fold higher than those with Restasis, respectively. It shows faster onset and higher stability, though ocular irritation and potential drug leakage remain possible adverse effects (26, 27).

CyclASol^®^ (Novaliq GmbH, Heidelberg, Germany), a 0.1% CsA semifluorinated alkane anhydrous solution contains no preservatives or surfactants, has high solubilizing capacity for hydrophobic drugs and good biocompatibility, and can reduce blurred vision as well as the risk of iatrogenic DED, with good tolerability (28–30). To overcome the rapid clearance of eye drops by tears, *in situ* gel systems can prolong drug release and improve bioavailability, though they have low oxygen permeability and may cause blurred vision (31).

CsA-loaded contact lenses (CL), including Brij-52 microemulsion, porous carrier, and amyloid nanofilm types, enhance bioavailability by 50%–82% compared with Restasis. They also prolong drug action and promote corneal healing without affecting optical performance (32, 33). Other novel formulations, including low-concentration CsA–trehalose nanoemulsion, lipid nanocapsules, cationic hyaluronic acid-coated formulations, and cyclodextrin-based oil-free CsA eye drops, have all demonstrated potential in improving bioavailability and tolerability (34–36).

### Clinical application of CsA

Currently, CsA alleviates inflammation and symptoms in moderate-to-severe DED and Sjögren’s syndrome (SS) (37–42), enhancing goblet cell density, mucin secretion, and tear film stability with efficacy comparable to corticosteroids (13, 43).

Multiple clinical studies have confirmed that CsA eye drops effectively improve ocular surface disease index (OSDI), tear film BUT, Schirmer’s test results, corneal fluorescein staining, and ocular discomfort (37–39). For example, the foundational 0.05% CsA emulsion (Restasis^®^) has been shown to improve Schirmer scores, BUT, and corneal staining, although it may require more than 6 months to demonstrate full efficacy. A multicenter study involving 240 Chinese patients showed that 0.05% CsA eye drops (II) (produced by Shenyang Xingqi Eye Medicine Co., Ltd., specification: 0.4ml: 0.2mg) administered twice daily for moderate-to-severe DED led to better total ocular scores than control after one week, with noticeable improvement within 1–2 months, and significantly superior outcomes compared with the control group after 3 months (23, 44). In a dose-ranging, randomized trial, the therapeutic effect of CsA generally emerges after several weeks of use, and study have shown that 0.05% and 0.1% concentrations achieve similar efficacy, with higher concentrations offering no obvious advantage (39).

In a retrospective analysis involving patients with meibomian gland dysfunction (MGD), CsA can reduce inflammation, improve eyelid margin scurf, telangiectasia, and meibum stasis, and enhance BUT and tear secretion (45). A Korean prospective, randomized, single-blinded, controlled clinical study demonstrated that 0.05% CsA nanoemulsion combined with warm compresses was more effective than sodium hyaluronate eye drops with warm compresses in treating MGD (46).

In cases of iatrogenic DED, such as post-LASIK and post-cataract surgery, CsA also shows good efficacy. One year after LASIK, CsA treatment restored Schirmer’s test and BUT to preoperative levels, improving DED indicators by approximately 30%, significantly better than the control group (47). In a prospective, open-label, randomized, controlled study among post-cataract surgery patients, three months of 0.05% CsA significantly improved Schirmer’s test results and BUT, and reduced dry eye symptom scores (48). A comparison between 3% diquafosol sodium and 0.05% CsA for post-cataract surgery DED found that the former improved tear secretion faster, while the latter was more effective in improving visual aberrations and required less frequent dosing (48).

In graft-versus-host disease (GVHD)-related DED, topical CsA improves dry eye symptoms, corneal sensitivity, tear evaporation rate, BUT, corneal staining, and conjunctival inflammatory cell infiltration (49). Preconditioning with topical CsA before hematopoietic stem cell transplantation (HSCT) can reduce postoperative dry eye severity, suggesting a preventive potential (50). An *in vivo* confocal microscopy study indicated that long-term use of preservative-containing medications in glaucoma patients often causes ocular surface damage; long-term CsA use can alleviate dry eye symptoms and prevent fibrosis (51).

Numerous randomized controlled trials worldwide have further confirmed the significant efficacy of CsA in moderate-to-severe DED. A multicenter study in China showed that combining 0.05% CsA with artificial tears for 8–12 weeks significantly improved OSDI score, corneal staining, tear secretion, and BUT, with an effective rate exceeding 70% (52). Phase III clinical trials abroad demonstrated that 0.1% CsA treatment for 6 months significantly improved ocular symptoms and reduced conjunctival HLA-DR expression (53). The latest approved 0.09% CsA nano-formulation has shown greater improvement in tear secretion and ocular surface symptoms in clinical studies (26). Clinical trials with different CsA concentrations indicate that 0.05% CsA is most effective in improving ocular symptoms, with higher concentrations showing no significant additional benefit (22).

### Adverse effects of CsA

The incidence and severity of adverse effects from topical CsA are largely concentration dependent. Low-concentration formulations show minimal systemic absorption and an excellent safety profile. The most frequently reported reactions are mild-to-moderate ocular irritation symptoms, such as stinging, burning, blurred vision, and conjunctival hyperemia, with no serious local or systemic adverse events observed (54–56). These irritation symptoms are often attributed not only to the active CsA molecule but also significantly to the formulation vehicle, such as the castor oil-based emulsion in traditional preparations, which can cause poor tolerability. In a clinical trial involving 1039 DED patients, CsA was well tolerated (57).

Reported adverse event rates vary across concentrations and formulations, with approximately 25.3% in the 0.05% group and 29% in the 0.1% group, predominantly involving mild-to-moderate eye pain or burning (58, 59). Differences in formulation may influence the incidence of adverse reactions.

Differences in formulation may influence the incidence of adverse reactions. Differences in vehicle composition may contribute to this variation. Transient stinging or burning upon instillation remains the main cause of treatment discontinuation in a minority of patients, highlighting the need for improved formulation tolerability. Nanoemulsion and cationic emulsion vehicles have been shown to lessen discomfort and enhance adherence compared with traditional oil-based preparations (60–62). Long-term follow-up has not identified any CsA-related systemic toxicity or sight-threatening adverse events, supporting its suitability for chronic use (63). Furthermore, combining CsA with artificial tears or short-term corticosteroids may reduce early irritation and improve compliance (64–66).

Regarding CL-based delivery systems, potential risks include bacterial resistance, ocular toxicity from hydrogel–drug interactions, and transient systemic exposure due to initial burst release (67). Emerging multifunctional biosensor–CL systems aim to reduce systemic toxicity, but remain in the research stage.

### Prospective

Currently, due to CsA’s strong hydrophobicity, the bioavailability of topical formulations remains low, limiting its clinical performance. Premature discontinuation often leads to symptom relapse, underscoring the need to define standardized treatment duration and tapering strategies. In addition, combination therapy has gained attention as a means to enhance efficacy and tolerability. Short-term topical corticosteroids can accelerate symptom relief in the early phase of CsA therapy by suppressing acute inflammation, while subsequent maintenance with CsA supports long-term control (68). Artificial tears remain indispensable as a complementary therapy to improve lubrication and patient comfort, and their concurrent use does not interfere with CsA’s pharmacodynamics (69). Secretagogues such as diquafosol or rebamipide may also provide synergistic benefits by improving mucin and aqueous tear secretion. Future research should clarify the optimal timing, sequence, and duration of these combination strategies to maximize therapeutic benefit while minimizing ocular irritation (70). Future investigations should also emphasize head-to-head comparisons between CsA and other immunomodulators (lifitegrast, tacrolimus), as well as the long-term impact of CsA on neurosensory recovery and ocular microbiome balance.

Future DED treatment strategies should be more individualized, taking into account different etiologies, disease courses, and severities, and formulating multi-level, long-term treatment plans. Incorporating biomarkers such as tear cytokine levels, HLA-DR expression, and patient genotypes into therapeutic decision-making could enable precision medicine approaches for CsA therapy, ultimately improving both efficacy and adherence in chronic DED management.

## Conclusion

In conclusion, with deeper understanding of DED pathophysiology and advances in delivery technologies, CsA’s application prospects in dry eye treatment are promising. Through continuous optimization of treatment regimens and innovative formulations, safer, more effective, and more personalized dry eye management can be achieved, ultimately improving patients` quality of life. Further comparative and mechanistic research is needed to determine CsA’s position among current and emerging therapies, guiding evidence-based, individualized treatment approaches.
